# Prevalence and risk factors of gastro-esophageal reflux disease among undergraduate medical students from a southern Indian medical school: a cross-sectional study

**DOI:** 10.1186/s13104-018-3569-1

**Published:** 2018-07-09

**Authors:** Ramachandran Arivan, Surendran Deepanjali

**Affiliations:** 1 Jawaharlal Institute of Postgraduate Medical Education and Research, 605006, Puducherry, India; 2 Department of Medicine, Jawaharlal Institute of Postgraduate Medical Education and Research, 605006, Puducherry, India

**Keywords:** Gastro-esophageal reflux disease, Prevalence, Medical students, Symptom score

## Abstract

**Objective:**

Gastro-esophageal reflux disease (GERD) affects all age groups, and various lifestyle as well as psychological factors are recognized as risk factors for GERD. Undergraduate medical students are exposed to lifestyle changes and psychological stressors. We aimed to study the prevalence of GERD among undergraduate students of a medical school in southern India in a cross-sectional survey using a validated symptom score.

**Results:**

A total of 358 undergraduate medical students participated in the study. There were 188 (52.5%) males and 170 (47.4%) females; the mean (SD) age of the participants was 20.3 (1.5) years. A total of 115 (31.2%) participants had at least one episode of heartburn per week, while 108 (30.1%) participants had at least one episode of regurgitation per week. Heartburn or regurgitation of at least mild severity was present in 115 (32.1%) and 108 (30.16%) of participants respectively. Based on the symptom score, a diagnosis of GERD was made in 18 (5.02%) students. Frequent consumption of carbonated drinks (OR = 3.63 [95% CI 1.39–9.5]; *P* = 0.008) and frequent consumption of tea or coffee (OR = 4.65 [95% CI 1.2–17.96]; *P* = 0.026) were significantly associated with a diagnosis of GERD.

**Electronic supplementary material:**

The online version of this article (10.1186/s13104-018-3569-1) contains supplementary material, which is available to authorized users.

## Introduction

Gastro-esophageal reflux disease (GERD) is defined as a condition that develops when reflux of stomach contents causes troublesome symptoms and/or complications [1]. Apart from the well- recognized complications like erosive esophagitis and Barrett’s esophagus, GERD has been linked to sleep disorders, metabolic syndrome, and coronary artery disease [2–4]. GERD affects almost all age groups starting from infancy. GERD was found to be more prevalent in the Europe and US, however recent studies from Asia point towards increasing prevalence in these regions also [5, 6]. A few studies from India indicate a prevalence of 7.6–18.7% [7–9].

Lifestyle factors like dietary habits, consumption of carbonated and alcoholic beverages and tobacco smoking have been investigated as risk factors for GERD. Importantly, psychological factors are linked to the illness experience and can modify subjective symptoms [10]. It is observed that the quality of life in those with GERD symptoms is lower when compared to the general population [11]. A unique combination of psychological factors and lifestyle changes occur when students enter undergraduate training in various disciplines. Studies show that medical undergraduate students especially have high levels of stress and perceived stress [12–14]. Various factors such as difficulty in coping up with the curriculum, staying away from home, and disordered or poor eating habits may contribute to the psychological distress. A limited number of studies are available regarding the prevalence and magnitude of GERD in college students [15, 16], medical students in particular [17, 18]. We aimed to study the prevalence of GERD and its associated risk factors among the undergraduate students of a medical school in India.

## Main text

### Materials and methods

#### Study participants

We conducted a questionnaire-based cross-sectional survey. The participants were undergraduate Bachelor of Medicine and Bachelor of Surgery (MBBS) students of Jawaharlal Institute of Postgraduate Medical Education and Research (JIPMER), a central government-run tertiary care institute located in Puducherry, southern India. In India, the MBBS program includes 4½ years of studies followed by compulsory internship for 1 year. The survey was conducted during August–September 2015. The study protocol was reviewed and approved by the Institute Ethics Committee (Human studies) at JIPMER (Ref. No. JIP/IEC/2015/19/698).

#### Procedure and instrument

The questionnaire was distributed to the students by one of the authors (RA) after the class hours after asking for their willingness to participate. Informed written consent was obtained from all the participants. We used a structured questionnaire for the purpose of the study (Additional file 1). Since English is the medium of instruction in Indian medical schools, the questionnaire was administered in English language in a paper format. The questionnaire comprised of basic demographic information followed by questions on lifestyle factors and medical and drug history. The final part was the assessment of GERD symptom score. The GERD symptom score used in this study has been previously validated in the Indian population [19]. This score is a self-reported grading of the frequency and severity of two main symptoms of GERD—heartburn and regurgitation (Additional file 1). Heartburn was defined as burning sensation or discomfort behind the breastbone in the chest and regurgitation was defined as a bitter or sour tasting food/liquid coming into the mouth. Final score for each symptom (heartburn and regurgitation) was obtained by multiplying the scores for severity and frequency. The total score was obtained by adding final scores of individual symptoms. Thus, the total scores could range from 0 to 18. Presence of GERD was defined as a score ≥ 4. GERD was further classified as mild, moderate, and severe based on total symptom scores of 4–8, 9–13, and 14–18 respectively.

#### Sample size calculation

Assuming the proportion of GERD in medical students to be 22% [20], a total of 358 participants were required to estimate this proportion with a relative precision of 20 and 95% confidence.

#### Statistical analysis

We used a statistical software package for analysis (Stata/IC 12.1 for Windows, StataCorp LP, College Station, Texas, USA). We summarized categorical variables as frequency with proportion [n (%)] and applied Chi square test for group comparisons. Normally distributed continuous variables were presented as mean (SD) and comparisons were done using independent t test. All tests were two-sided, and we considered *P* < 0.05 statistically significant.

### Results

**Fig. 1:**
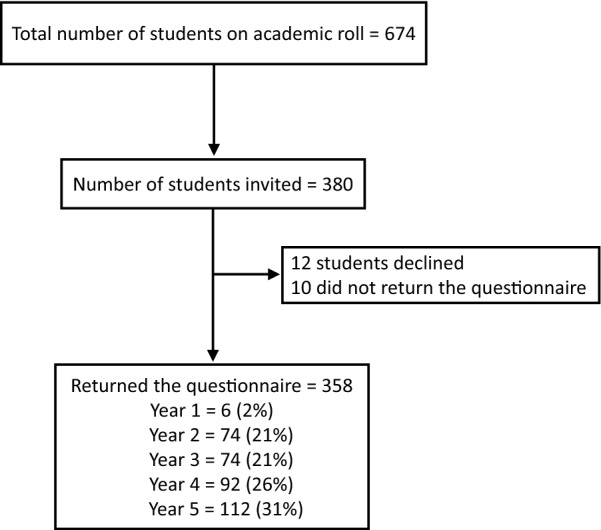
Recruitment of study participants

During the study period, a total of 674 undergraduate MBBS students were on the academic rolls. Of them, 358 students completed the questionnaire. Figure 1 depicts the recruitment of participants into the study. There were 188 (52.5%) males and 170 (47.4%) females. The mean (SD) age of the participants was 20.3 (1.5) years. Data on body-mass index (BMI) was available for 306 (85.4%) participants and the mean (SD) BMI was 22.27 (3.4) kg/m^2^.

#### Medical and drug history

Of the 358 participants, 52 (14.5%) reported at least one medical illness. Of them, 6 indicated that they had ‘GERD’, 1 had ‘gastritis’ and 1 had ‘peptic ulcer’. A diagnosis of bronchial asthma was reported by 15 participants. There were 105 (29.3%) participants who were already taking antacids, of whom 4 were taking it on a daily basis. Frequent (> 2 times a week) use of over the counter analgesics like paracetamol, diclofenac, and ibuprofen was reported by 5 participants.

#### GERD symptom scores and association with lifestyle factors

**Fig. 2:**
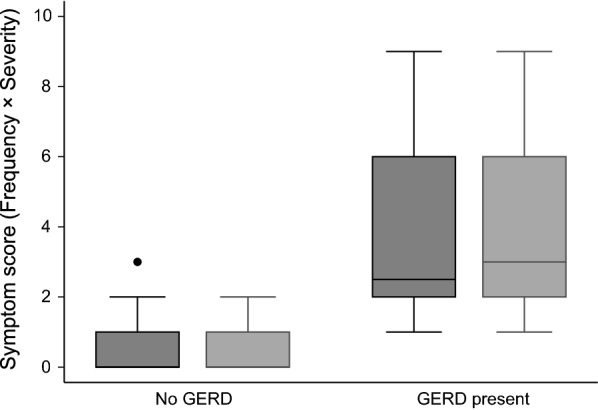
Box-whisker plot depicting distribution of symptom scores for heartburn (black) and regurgitation (gray) among students with and without GERD

Of the 358 participants, 193 (53.9%) did not report any symptoms of heartburn or regurgitation. A total of 115 (31.2%) participants had at least one episode of heartburn per week, while 108 (30.1%) participants had at least one episode of regurgitation per week. Similarly, heartburn or regurgitation of at least mild severity was present in 115 (32.1%) and 108 (30.16%) of participants respectively. A diagnosis of GERD was made in 18 (5.02%) subjects who had a final symptom score of 4 or more. Of them, 11 had mild, 5 had moderate, and 2 had severe GERD. Distribution of symptom scores for heartburn and regurgitation among students with and without GERD is shown in Fig. 2.

There was no significant association between prevalence of GERD and gender, BMI, or the year of study. However, students with any grade of heartburn or regurgitation had a significantly higher BMI compared to those with no symptoms (22.74 ± 3.4 vs. 21.83 ± 3.3 kg/m^2^; *P *= 0.018). Among the lifestyle factors, frequent consumption of carbonated drinks (OR = 3.63 [95% CI 1.39–9.5]; *P *= 0.008) and tea or coffee (OR = 4.65 [95% CI 1.2–17.96]; *P *= 0.026) were significantly associated with a diagnosis of GERD. No statistically significant association was observed between other lifestyle factors and GERD (Table 1).

**Table 1:** Comparison of lifestyle factors between undergraduate medical students with and without GERD

Lifestyle factor	GERD present (N = 18)	No GERD (N = 340)	*P* value
Non-vegetarian diet	15 (83.3)	288 (84.7)	0.875
Frequent night snacks	7 (38.8)	83 (24.4)	0.17
Frequently skips breakfast	9 (50)	153 (45)	0.67
Frequent consumption of carbonated drinks	10 (55.5)	87 (25)	0.005
Drinking > 3 cups of tea or coffee per day	3 (16.6)	14 (4.1)	0.015
Less than 6 h sleep at night	10 (55.5)	140 (41.1)	0.23
Consumption of alcohol	4 (22.2)	49 (14.4)	0.36
Smoking	1 (5.5)	8 (2.3)	0.39
Frequently chewing tobacco products	0 (0)	1 (0.3)	0.82
Frequent use of analgesics	4 (22.2)	99 (29.1)	0.53
Physical exercise at least 5 times per week	4 (22.2)	90 (26.4)	0.69

### Discussion

We found that 30% of undergraduate students of a medical school in southern India had at least one episode of heartburn or regurgitation in a week, and 5% of students qualified for a diagnosis of GERD defined by a symptom score ≥ 4. In a previous study on GERD symptoms in undergraduate students from another medical school in southern India, the authors found a similar prevalence of weekly symptoms (24%) [17]. However, a study from Karachi, Pakistan reported a lower prevalence of weekly symptoms of about 7% among medical students [18]. No data on severity of symptoms was reported in these two studies, precluding estimation of the true prevalence of GERD.

Previous studies from India using the same questionnaire in different study populations have reported a higher prevalence of GERD. A study on employees of a government hospital in northern India found a prevalence of 16.2% [7]. Another study conducted in Ladakh, which is a high altitude area, reported a prevalence of 18.7% [8]. There could be two possible reasons for the lower prevalence of GERD in the present study. First, the mean age of participants in the previous studies from India was higher by about 2 decades. Increasing prevalence of GERD symptoms with age has been observed in some studies, though not in others [8, 21]. Second, previous studies had included a heterogeneous adult population, whereas we studied a more homogeneous group. GERD belongs to a group of gastrointestinal disorders in which the perception of symptoms and its severity has been shown to be modified by stress and stress-related personality characteristics. Moreover, studies reveal that medical students often encounter several barriers in seeking help for physical and mental health problems [22, 23]. A recent study from our institution showed that a significant proportion of undergraduate students had fear of side-effects of treatment and negative academic impact if they sought medical attention, and hence were hesitant to do so [23]. Also, more than 75% of them resorted to self-medication for their physical illnesses. Likewise, in another study from southern India, about 48% of undergraduate medical students took self-medications for GERD symptoms especially before their exams [17]. It is to be noted that about 30% of students were taking some form of antacids, although we did not collect information on whether this was over-the-counter use.

We found that the prevalence of GERD symptoms was more in students with a higher BMI. A dose dependent increase in GERD symptoms with increasing BMI was observed in the HUNT study, a prospective population-based cohort study conducted in Nord-Trøndelag County, Norway [21]. A recent meta-analysis on lifestyle interventions effective in reducing GERD symptoms concluded that weight loss and tobacco cessation were the two effective measures [24]. This is especially relevant for the student population since disordered eating habits and lack of adequate physical exercise could potentially lead to weight gain. We observed that 35% of students did not engage in any form of physical exercise.

We found that frequent consumption of carbonated beverages was a risk factor for GERD. Some physiological studies indicate that certain foods and beverages can relax the lower esophageal sphincter and cause a drop in esophageal pH [25, 26]. However, a systematic review on the effects of carbonated drinks on GERD found no direct evidence that these drinks promote or exacerbate GERD [27]. These beverages are known to have other deleterious health effects like weight gain, and therefore it is prudent to discourage their frequent consumption [28, 29]. Frequent consumption of tea or coffee was also associated with GERD. Drinking two cups of tea per day was a risk factor for GERD in Syrian undergraduate and graduate students [16]. Similarly, a study on men from Taiwan found that drinking tea ≥ 4 days a week was associated with asymptomatic erosive esophagitis [30]. Students with GERD symptoms could be advised to observe if tea or coffee exacerbate their symptoms so that their intake can be limited.

The present study has the merit that we used a standardized, validated questionnaire for the diagnosis of GERD. This ensures that mild, infrequent symptoms which represent physiological gastro-esophageal reflux are not misclassified as disease. Further, we asked the students to comment on current episodes of GERD symptoms and lifestyle, thereby minimizing recall bias.

### Conclusions

In conclusion, while about one-third of undergraduate medical students had varying degree of symptoms suggestive of reflux disease, about 5% had GERD. Frequent consumption of carbonated beverages and tea or coffee was associated with GERD. Further studies are needed to explore the role of physical and psychological factors in the students’ illness experience.

## Limitations

There were only 6 participants belonging to the 1st year of medical school. It is possible these students may have higher prevalence of GERD symptoms since they would have been least adjusted to the physical and psychosocial challenges of having started the college life. Many of them had started their hostel life recently and might have had difficulty in adjusting to the changed food options. Also, we did not collect data on certain other lifestyle factors like interval between dinner and night sleep as well as head elevation during sleep, which could have a bearing on symptoms.

## Additional file

**Additional file 1.** Study questionnaire and GERD symptom score.

## Abbreviations

GERD: gastro-esophageal reflux disease
BMI: body-mass index
