# Supplementary material for: Prevalence and risk factors of gastro-esophageal reflux disease among undergraduate medical students from a southern Indian medical school: a cross-sectional study
Source: BMC Res Notes. 2018 Jul 9;11:448. doi: 10.1186/s13104-018-3569-1 (PMC6038284; doi:10.1186/s13104-018-3569-1)
Supplement: Supplementary file 1 — Additional file 1. Study questionnaire and GERD symptom score. [file 13104_2018_3569_MOESM1_ESM.docx]

**QUESTIONNAIRE**

**Prevalence of gastroesophageal reflux disease among medical students of JIPMER**

Name:

Age:

Sex: Male/Female

M.B.B.S batch:

Address:

Phone no. :

Body weight:

Height:

Following questions are regarding your dietary habits. Please shade the appropriate response

1. Are you a vegetarian or a non- vegetarian?

☐ Veg ☐ Non-veg

1. If you are a Non-vegetarian, how often do you eat Non-veg?

☐ Daily ☐ A few times in a week ☐ A few times in a month

1. How often do you have food from restaurants?

☐ Daily ☐ A few times in a week ☐ A few times in a month

1. How frequently do you consume aerated drinks?

☐ Never ☐ Infrequently ☐ Frequently

1. How often do you consume tea/coffee?

☐ Never ☐ 1-3 cups per day ☐ >3 cups/day

1. How often do you have a mid-night snack?

☐ Never ☐ Infrequently ☐ Frequently

1. Do you skip your breakfast?

☐ Skip everyday ☐ Skip frequently ☐ Skip occasionally ☐ Never skip

1. How long do you sleep at night?

☐ 1-3hours ☐ 3-6hours ☐ >6 hours

1. Smoking habits [Pack-year = (No. of cigarette packets per day)×No. of years of smoking]

☐ Non-smoker ☐ Ex-smoker (No. of Pack-years____) ☐ Smoker (No. of Pack- years____)

1. Do you use alcoholic drinks?

☐ Never ☐ Occasionally ☐ Frequently ☐ Daily

If you use alcohol, what type of alcohol do you consume and approximately quantify the amount consumed.

1. Do you chew tobacco or nicotine products?

☐ Never ☐ Occasionally ☐ Frequently ☐ Daily

1. Do you engage in physical exercise?

If yes, the frequency is

☐ Everyday ☐ At least 5 times per week ☐ <5 times per week

Mode of exercise

☐ walking briskly ☐ Jogging ☐ Going to the gym

Duration of exercise

☐ >30 minutes ☐ 30 minutes ☐ <30 minutes

1. Have you been diagnosed with any medical illness in the past?

☐ Yes ☐ No

If yes, please mention the diagnosis.

1. Are you taking any drugs routinely (allopathic/alternative medicines) or frequently? If yes, please mention the drug.
2. Do you take over-the-counter analgesics like paracetamol, diclofenac (Voveran), ibuprofen (Brufen) etc.?

☐ Yes ☐ No

1. If yes, how frequently?

☐ More than 2 times per week ☐ Less than 2 times per week ☐ Occasionally ☐ Only when prescribed for any fever/pain

1. Do you have symptoms like heartburn or regurgitation?

☐ Yes ☐ No

If yes, what relieves the symptoms?

☐ Relieves spontaneously ☐ Medication ☐ Eating ☐ Others (Please mention)

1. Do you take any antacids (Gelusil, Mucaine gel)/proton pump-inhibitors (Omeprazole, rabeprazole, pantoprazole)/ H_2_bloking drugs (Ranitidine, cimetidine)?

☐ Yes ☐ No

1. If yes, how frequently?

☐ Daily ☐ SOS whenever symptomatic ☐ Never

**GERD symptom score**:

Please grade the severity and frequency of heartburn and/or regurgitation of food, if any. Please tick the appropriate box for each.

Heartburn is “felt as a burning sensation in the chest”.

Regurgitation is “a feeling of sour food/liquid coming into the throat”.

**Severity Grading**

| **Grading** | **Symptom** | |
| --- | --- | --- |
|  | **Heartburn** | **Regurgitation** |
| **Grade 0:** No symptoms |  |  |
| **Grade 1:** Mild symptoms with spontaneous remission.  No interference with normal activity and sleep. |  |  |
| **Grade 2:** Moderate symptoms with spontaneous but slow remission. Mild interference with normal activity and sleep. |  |  |
| **Grade 3:** Severe symptoms without spontaneous remission.  Marked interference with normal activity and sleep. |  |  |

**Frequency grading**

| **Grading** | **Symptom** | |
| --- | --- | --- |
|  | **Heartburn** | **Regurgitation** |
| **Grade 0:** Absent |  |  |
| **Grade 1:** Occasional (<2 days per week) |  |  |
| **Grade 2:** Frequent (2–4 days per week) |  |  |
| **Grade 3:** Very frequent (>4 days per week) |  |  |
